# Supplementary material for: Bacterial Outer Membrane Vesicles as a Platform for the Development of a Broadly Protective Human Papillomavirus Vaccine Based on the Minor Capsid Protein L2
Source: Vaccines (Basel). 2023 Oct 11;11(10):1582. doi: 10.3390/vaccines11101582 (PMC10611245; doi:10.3390/vaccines11101582)
Supplement: Supplementary file 1 [file vaccines-11-01582-s001.zip › vaccines-2628281-supplementary.pdf]

Supplementary Material

Bacterial Outer Membrane Vesicles as a Platform for the Development of a Broadly Protective Human Papillomavirus Vaccine Based on the Minor Capsid Protein L2

**Silvia Tamburini [1], Yueru Zhang [2], Assunta Gagliardi [3], Gabriele Di Lascio [3], Elena Caproni [3], Mattia Benedet [3], Michele Tomasi [1], Riccardo Corbellari [1], Ilaria Zanella [1], Lorenzo Croia [1], Guido Grandi [1,*], Martin Müller [2]
and Alberto Grandi [3,4]**

* Correspondence: guido.grandi@unitn.it

Supplementary Figures

Figure S1

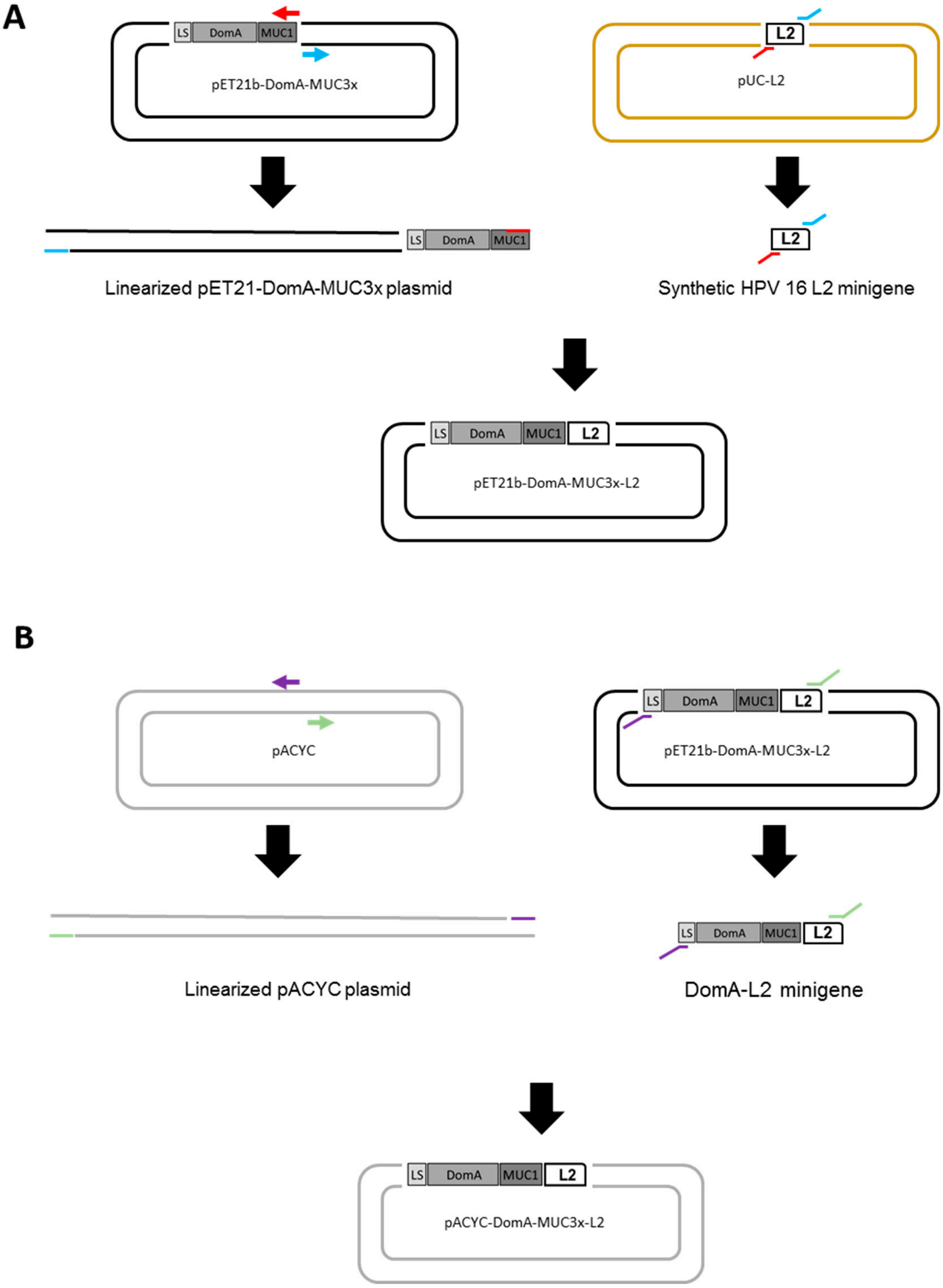

Figure S1. Cloning strategy of HPV16 L2 fusion. (A) Cloning of fHbp-DomA-MUC3x-$L2_{16}$ gene in pET. pET_Nm-fHbpDomA_MUC1[13] was linearized with two divergent primers (see Table 1) at the C-terminus of the MUC1 epitope. In parallel the $L2_{16}$ coding sequence was amplified from pUC plasmid carrying the synthetic DNA encoding the $L2_{16}$ epitope. Finally, the linearized pET-fHbp-DomA-MUC3x and the amplified $L2_{16}$ minigene were combined to

generate plasmid pET-fHbp-DomA-MUC3x-$L2_{16}$. (B) Cloning of fHbp-DomA-MUC3x-$L2_{16}$ gene in pACYC. The low copy number plasmid pACYC was linearized with primers annealing downstream from the T7 promoter. In parallel the fHbp-DomA-MUC3x-$L2_{16}$ gene was amplified from pET-fHbp-DomA-MUC3x-$L2_{16}$. The fHbp-DomA-MUC3x-$L2_{16}$ fragment and the linearized pACYC were mixed together to generate pACYC-DomA-MUC3x-$L2_{16}$. The cloning strategy just described was exactly the same used also to generate all the other L2 fusions described in the text.

Figure S2

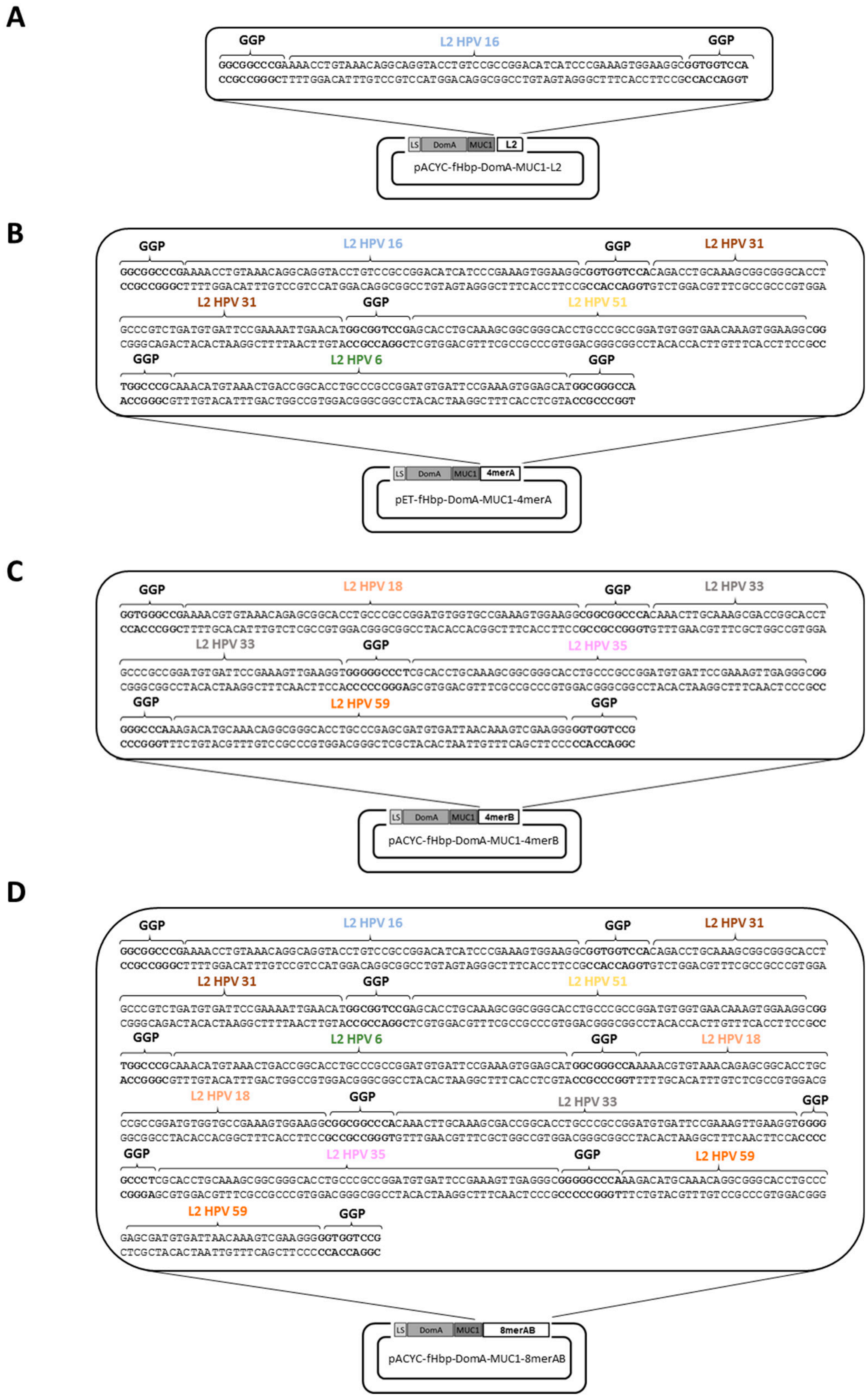

Figure S2. Schematic representation of the plasmids coding for the HPV L2 fusions.

Figure S3

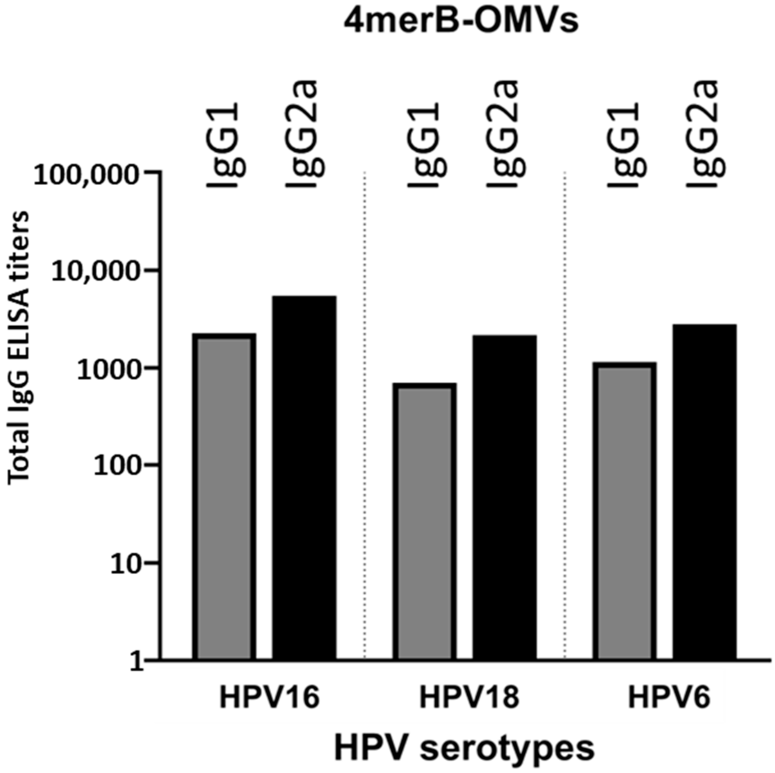

Figure S3. Antigen-specific titers of IgG isotype. Sera from mice immunized i.p. with three doses of 4merB-OMVs were pooled and IgG1 and IgG2a titers were analyzed by ELISA coating the plate with HPV16, HPV18 and HPV6 L2 peptides (0.5 μg/well).

Figure S4

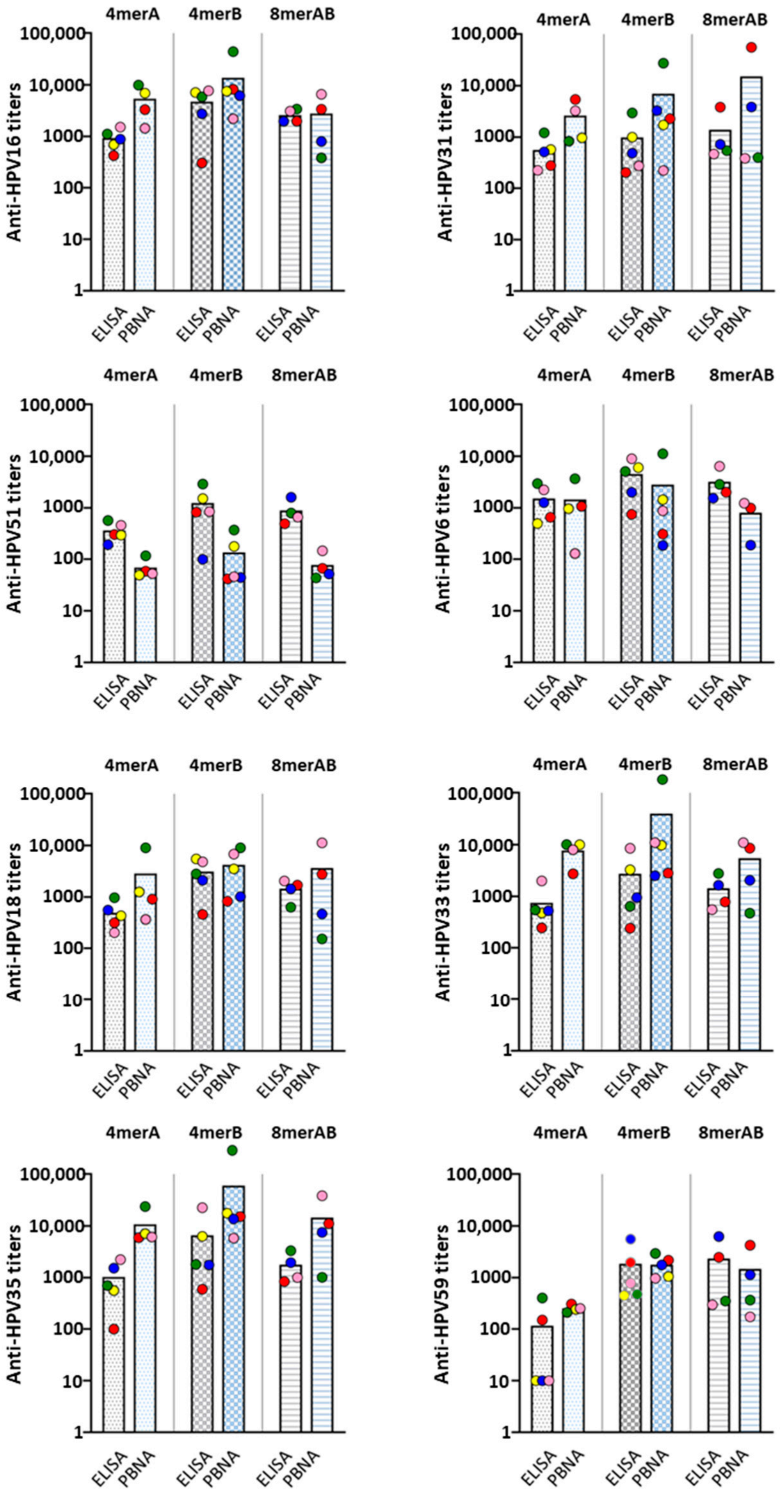

Figure S4. ELISA and neutralization (PBNA) titers induced by OMV-based vaccines. ELISA (Grey) and PBNA (Light blue) neutralization titers of the sera from mice immunized with 4merA-OMVs, 4merB-OMVs and 8merAB-OMVs (same sera described in Figures 2 and 3). Each graph groups the ELISA and neutralization titers against one of the selected

HPV serotypes, elicited by the three vaccine formulations. To follow the correlation between the ELISA and the neutralization titers induced by each vaccine formulation, to each mouse serum has been assigned a circle with a different color code. ELISA titers correspond to the serum dilution that gives an OD_{405} value = 1.5 and the PBNA EC_{50} value calculated as the titer of serum that could neutralize half of the pseudovirus. Both titers are expressed in logarithmic scale. Depending upon serum availability, four or five sera from each group were analyzed. Graphs were made with GraphPad 8 software.

Figure S5

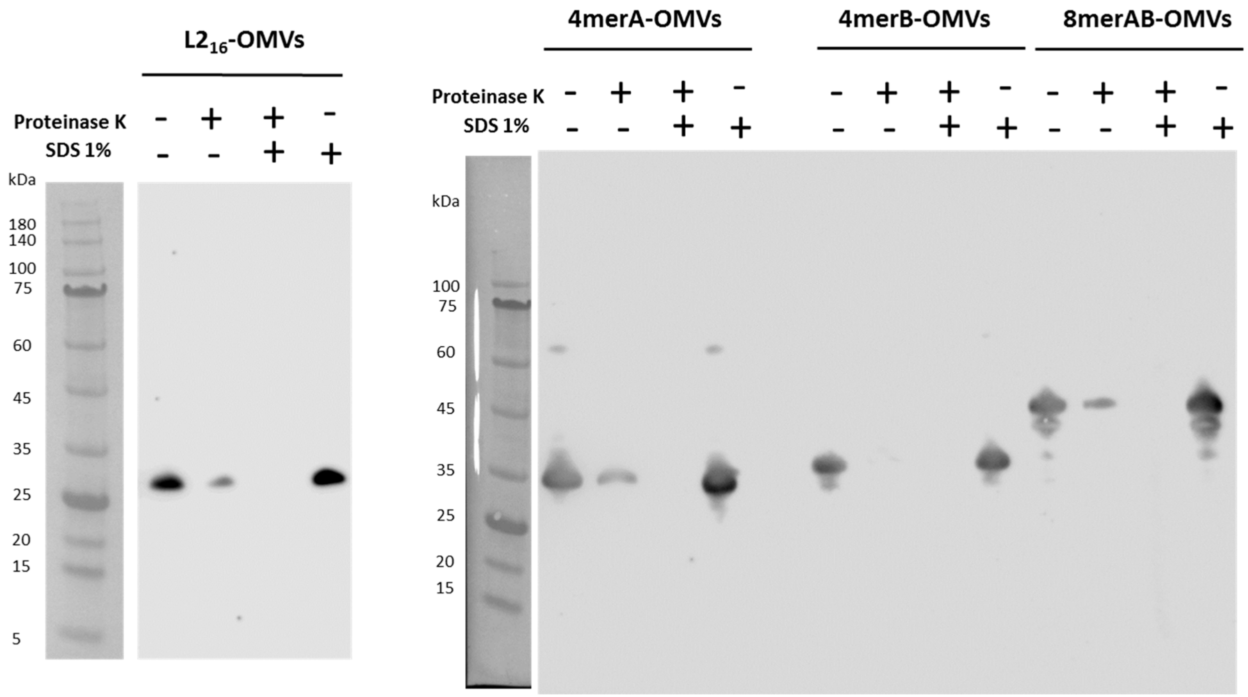

Figure S5. Uncropped Western Blot membranes presented in Figure 1D.

Table S1. Primers used for gene cloning strategies.

Name	Sequence	Use
pET_F	CATCACCATCACCATCACGATTACA	Linearization plasmid pET
MUC3X_R	ATGCGCCGGCGGCGC	Linearization plasmid pET
L2_F_pET	GCGCCGCCGGCGCATGGCGGCCCGAAAACC	Cloning L2, 4merA and 8merAB
L2_R_pET	GATGGTGATGGTGATGTTATGGACCACCGCCTTCCA C	Cloning L2
4merA_R_pET	GATGGTGATGGTGATGTTATGGCCCGCCATGCTCCA	Cloning 4merA
4merB_F	GCGCCGCCGGCGCATGGTGGGCCGAAAACGTGT	Cloning 4merB
4merB_R_pET	GATGGTGATGGTGATGTTACGGACCACCCCCTTCGA C	Cloning 4merB and 8merAB
PACYC_F	AGCCAGGATCCGAATTCGAGC	Linearization plasmid pACYC
PACYC_R	GGTATATCTCCTTATTAAAGTTAAAC	Linearization plasmid pACYC
fHDa_F_pACYC	ATAAGGAGATATACCGTGAATCGAACTGCC	Cloning entire constructs (fHDa-MUC3x + epitopes) in pACYC
L2_R_pACYC	ATTCGGATCCTGGCTTTATGGACCACCGCCTTCCAC	Cloning L2
4merB_R_pACYC	ATTCGGATCCTGGCTTTACGGACCACCCCCTTC	Cloning 4merB and 8merAB
T7-P	TAATACGACTCACTATAGGG	Sequencing in pET
T7-T	GCTAGTTATTGCTCAGCGG	Sequencing in pET
PACYC-up1	GGATCTCGACGCTCTCCCT	Sequencing in pACYC
PACYC-down1	GATTATGCGGCCGTGTACAA	Sequencing in pACYC
